# Compound Odontoma Removed by Endoscopic Intraoral Approach: Case Report

**DOI:** 10.3390/dj9070081

**Published:** 2021-07-07

**Authors:** Masakazu Hamada, Rena Okawa, Kyoko Nishiyama, Ryota Nomura, Narikazu Uzawa, Kazuhiko Nakano

**Affiliations:** 1 Department of Oral and Maxillofacial Surgery II, Osaka University Graduate School of Dentistry, Osaka 565-0871, Japan; hmdmskz@dent.osaka-u.ac.jp (M.H.); k-nishiyama@dent.osaka-u.ac.jp (K.N.); uzawa@dent.osaka-u.ac.jp (N.U.); 2 Department of Pediatric Dentistry, Osaka University Graduate School of Dentistry, Osaka 565-0871, Japan; rnomura@dent.osaka-u.ac.jp (R.N.); nakano@dent.osaka-u.ac.jp (K.N.)

**Keywords:** compound odontoma, odontogenic tumor, endoscopic intraoral approach

## Abstract

A 12-year-old Japanese boy was referred to our hospital for evaluation of a radiopaque area on the left side of the mandible. Radiographic and computed tomographic examinations revealed a radiopaque lesion located on the lingual side, along with permanent tooth eruption. Several small tooth-like structures were noted within the lesion and the mandibular left second premolar was inclined in a mesial direction. An odontoma was clinically diagnosed and surgical removal by an endoscopic intraoral approach under general anesthesia was planned. Reports of oral surgery using an endoscopic approach have been presented, though none for an odontoma. With the expectation that removal of the odontoma would improve dentition in this case, we planned future management. A minimally invasive surgical removal procedure by an endoscopic intraoral approach from the lingual side was performed and good early recovery was noted. The resected tumor consisted of several small tooth-like structures. Histopathological diagnosis was a compound odontoma. One-year follow-up findings showed that the post-surgical course was good.

## 1. Introduction

Odontomas are one of the most common benign odontogenic tumors that occur in the jaw and are composed of enamel, dentin, cementum, and pulp tissue [1,2]. The 4th edition of the World Health Organization’s Classification (WHO classification) of odontogenic tumors published in January of 2017 divides these tumors into complex and compound odontoma [3], which are usually asymptomatic lesions found incidentally during routine radiography [4]. Analyses of odontoma cases in Japan have shown that about half of the patients are between the ages of 10 and 19, with the detection rate for those under the age of 10 reported to be about 10% [5,6]. If no effect on the dentition is observed, treatment may be difficult depending on the site of occurrence. In recent years, endoscopes have come to be used in a variety of areas [7,8]. It has also been used in the field of oral surgery [9,10,11,12,13]. However, there are no reports of its use for odontomas. Here, we report a case of compound odontoma that developed in the mandible of a child and was removed from the lingual side by use of an endoscopic intraoral approach.

## 2. Case Report

**Figure 1:**
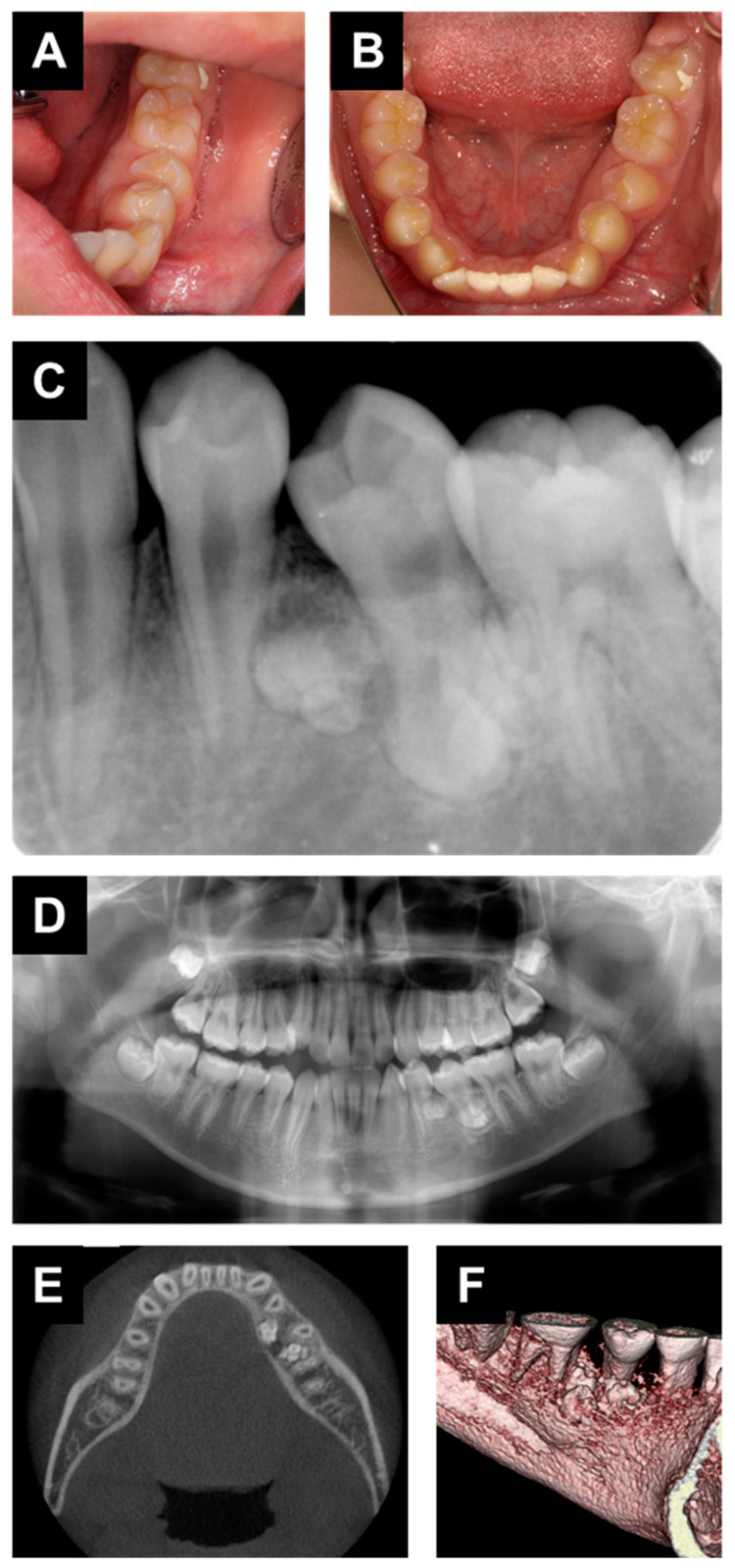
(**A**,**B**) Intraoral photographs taken at the initial visit. (**C**) Panoramic radiography, (**D**) periapical radiography, and (**E**) computed tomography images. (**F**) Three-dimensional construction of affected region.

A 12-year-old boy was referred to the Department of Oral and Maxillofacial Surgery at Osaka University Dental Hospital for evaluation of a radiopaque area on the left side of the mandible. There was no special mention of systemic history and the patient reported no symptoms in the affected area. An intraoral examination revealed no obvious gingival swelling around the left mandibular region (Figure 1A), though the mandibular left second premolar was inclined in a mesial direction (Figure 1B). Panoramic and periapical radiography findings showed a radiopaque area around the root apices of the left mandibular first premolar, left mandibular second premolar, and left mandibular first premolar (Figure 1C,D). Computed tomography (CT) results showed several small tooth-like structures within the lesion located on the lingual side (Figure 1E,F), and an odontoma was clinically diagnosed.

**Figure 2:**
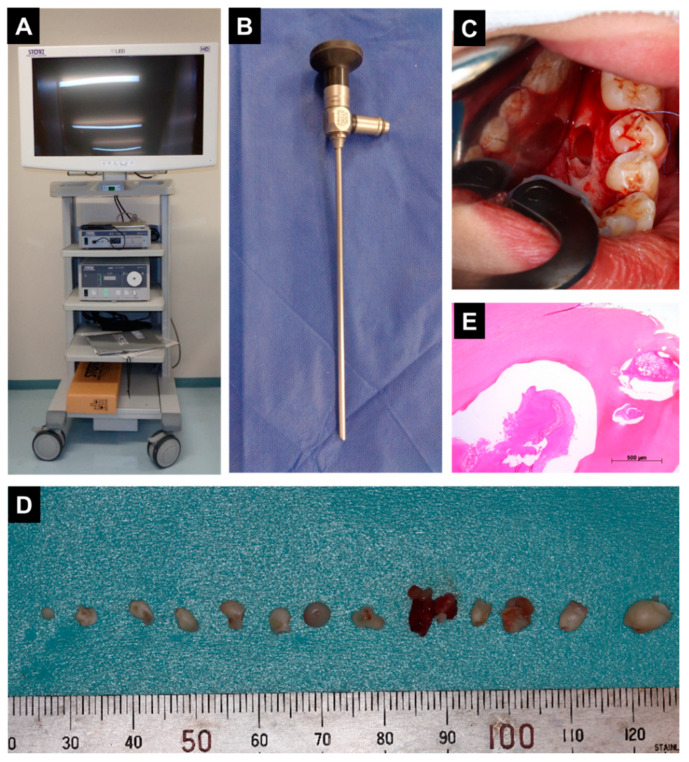
(**A**,**B**) The endoscope used in the surgery, (**C**) Intraoperative appearance after removal of tumor. (**D**) Excised surgical specimen showing tumor mass. (**E**) A histological examination of the specimen showed irregularly sized vascular spaces surrounded by dense connective tissue (H and E staining).

Under general anesthesia, minimally invasive surgical removal with an endoscopic intraoral approach from the lingual side was performed (Figure 2A–C). Since it was difficult to see the lesion directly or with a dental mirror from the lingual side, we decided to use an endoscope for this surgery. We used the KARL STORZ Endoskope (KARL STORZ, Tuttlingen, Germany). The endoscope was used to ensure that the lesion was removed. The resected tumor was found to consist of several small tooth-like structures (Figure 2D). In histopathological analysis findings, hematoxylin and eosin staining of the tooth-like structures showed them to be composed of dentin and cementum with centrally located loose fibrous tissue, considered to be pulp tissue (Figure 2E). Histopathological diagnosis was a compound odontoma.

**Figure 3:**
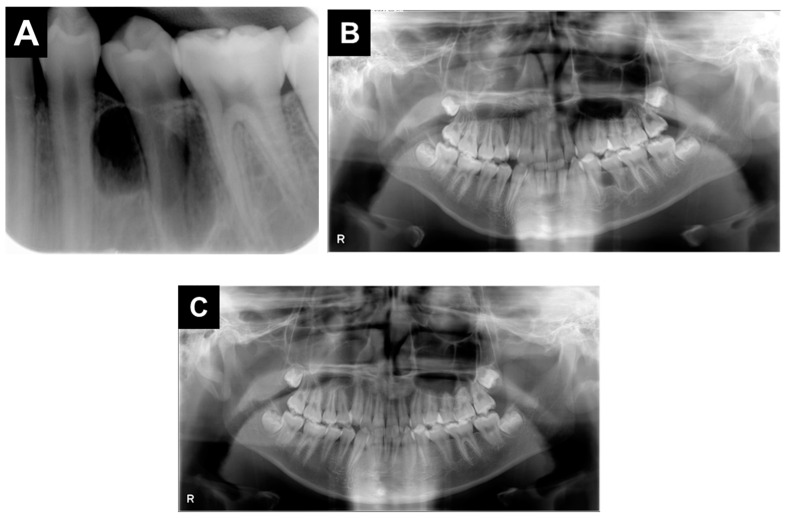
(**A**) Periapical and (**B**) panoramic radiography images after surgery obtained at follow-up examination. (**C**) Panoramic radiography image at 1-year follow-up.

Adjacent tooth roots showed no problems (Figure 3A,B). One year after removal of the odontoma, no recurrence was seen and though dentition improvement was expected, that was not noted (Figure 3C). Furthermore, devitalization of premolars, such as root resorption, tooth discoloration, or mobility, was not observed. The parents and patient did not wish to continue treatment for dentition or occlusion from that time.

## 3. Discussion

Odontomas are one of the most common benign odontogenic tumors that occur in the jaw, and can be divided into complex and compound type [1,2,3]. Among odontogenic tumors, the frequency of ameloblastomas has been reported to be highest at 45.2%, followed by odontomas at 24.9%, with the incidence rate of complex odontoma 9.7% and that of complex odontoma 15.3% [1]. These tumors are usually asymptomatic lesions found incidentally during a routine radiography examination, and standard treatment of an odontoma associated with soft tissue is complete removal so as to avoid recurrence [2,4]. In the present case, complete removal was the first choice.

Complications associated with an odontoma include eruption disturbance and tooth malposition, while these tumors have also been linked to root resorption of adjacent teeth [14,15]. In the present patient, the odontoma did not cause any eruption problems, and was noticed incidentally in panoramic radiography and periapical radiograph results. Although there was no problem with tooth eruption, the lesion was located on the lingual rather than buccal side, thus it was necessary to consider an approach that would not damage adjacent teeth during extraction. Because of the location between the mandibular left first premolar and mandibular left second premolar, the mandibular left second premolar showed a partial curvature and centrifugal inclination of the root, which caused its tooth crown to be slanted in a proximal direction.

Teeth that erupt in a low position may have increased risk of dental caries or periodontal problems due to difficulties with oral hygiene status, and can also cause various other problems. Such a condition can be prevented by early detection and extraction for avoiding abnormalities in the direction of tooth eruption. Diagnosis based on panoramic radiography results is important when malalignment is observed. However, extraction of an odontoma located between adjacent roots of teeth under formation is risky. Therefore, it is important to detect such a lesion early and consider the timing of extraction while observing the eruption of permanent teeth. In the present case, we planned dentition management with the expectation that removal of the odontoma would improve the condition.

A minimally invasive surgical approach is important for function preservation, as well as patient satisfaction and early recovery. In the present case, careful removal of the tumor in a manner to spare bone not damage the premolars under root formation was considered important. Endoscopic technology, typically employed for a minimally invasive procedure, is widely used with various surgical techniques to reduce tissue invasion during surgery and minimize bleeding [7,8]. In the field of oral surgery, it has been used for cases with fractures of the condylar neck, extraction of deep impacted teeth, and molar apicoectomy procedures [9,10,11,12,13]. On the other hand, there are no known reports regarding use of an endoscopic approach for removal of an odontoma. By using an endoscope, cases typically requiring an extraoral approach can be treated with an intraoral approach, as the technique is designed to reduce the possibility of skin scarring and facial nerve damage as much as possible. Benefits of endoscopically guided surgery in the dental field include minimal invasiveness, avoidance of facial incisions, and reduced amounts of bone loss, bleeding, and nerve and soft tissue damage (Table 1).

In the present case, CT results showed several small tooth-like structures within the lesion and their location on the lingual side. A method performed under direct vision is usually selected for removal of an odontoma, though limitations include difficulty with visualizing the lingual side or near the tooth root, which subsequently increases operative time and risk of iatrogenic damage, such as damage to neurovascular structures or excessive bone removal. Since it was difficult to see the lesion directly from the lingual side or with a dental mirror, we decided to use an endoscope for this procedure. With development of intraoral endoscopy techniques, more conservative bone removal has become possible, and we were able to avoid the risk of root damage in the adjacent tooth and confirm that the lesion had been definitely removed.

## Figures and Tables

**Table 1:** Advantages of endoscopic intraoral approach.

Minimally invasive Facial incisions avoided Reduced bone loss Reduced bleeding Reduced nerve damage Reduced soft tissue damage

## Data Availability

Not applicable.
